# Antimicrobial Properties and Therapeutic Potential of Bioactive Compounds in *Nigella sativa*: A Review

**DOI:** 10.3390/molecules29204914

**Published:** 2024-10-17

**Authors:** Munawar Abbas, Mayank Anand Gururani, Amjad Ali, Sakeena Bajwa, Rafia Hassan, Syeda Wajiha Batool, Mahreen Imam, Dongqing Wei

**Affiliations:** 1 College of Food Science and Engineering, Henan University of Technology, Zhengzhou 450001, China; shigrimunawar3@gmail.com; 2 Biology Department, College of Science, UAE University, Al Ain P.O. Box 15551, United Arab Emirates; 3 Department of Sustainable Crop Production, Università Cattolica del Sacro Cuore, Via Emilia Parmense 84, 29122 Piacenza, Italy; amjadparachinar@gmail.com; 4 Department of Medical Laboratory Technology, Riphah International University, Faisalabad 44000, Pakistan; 5 Department of Biological Sciences, Pakistan Institute of Engineering and Applied Sciences, Islamabad 45650, Pakistan; rafia.gcuf11@gmail.com; 6 Department of Biotechnology, National Institute for Biotechnology and Genetic Engineering, Faisalabad 38000, Pakistan; 7 Department of Biotechnology, Government College University, Faisalabad 38000, Pakistan; 8 State Key Laboratory of Microbial Metabolism, School of Life Sciences and Biotechnology, and Joint Laboratory of International Cooperation in Metabolic and Developmental Sciences, Ministry of Education, Shanghai Jiao Tong University, 800 Dongchuan Road Shanghai, Minhang District, Shanghai 200240, China; 9 Zhongjing Research and Industrialization Institute of Chinese Medicine, Zhongguancun Scientific Park, Meixi, Nanyang 473006, China; 10 Henan Biological Industry Group, 41, Nongye East Rd, Jinshui, Zhengzhou 450008, China; 11 Peng Cheng National Laboratory, Vanke Cloud City Phase I Building 8, Xili Street, Nashan District, Shenzhen 518055, China

**Keywords:** *Nigella sativa*, antimicrobial, thymoquinone, bioactive compounds, microorganisms, antibiotic, medicinal plant

## Abstract

*Nigella sativa* (*N. sativa*; Ranunculaceae), commonly referred to as black cumin, is one of the most widely used medicinal plants worldwide, with its seeds having numerous applications in the pharmaceutical and food industries. With the emergence of antibiotic resistance in pathogens as an important health challenge, the need for alternative microbe-inhibitory agents is on the rise, whereby black cumin has gained considerable attention from researchers for its strong antimicrobial characteristics owing to its high content in a wide range of bioactive compounds, including thymoquinone, nigellimine, nigellidine, quercetin, and O-cymene. Particularly, thymoquinone increases the levels of antioxidant enzymes that counter oxidative stress in the liver. Additionally, the essential oil in *N. sativa* seeds effectively inhibits intestinal parasites and shows moderate activity against some bacteria, including *Bacillus subtilis* and *Staphylococcus aureus*. Thymoquinone exhibits minimum inhibitory concentrations (MICs) of 8–16 μg/mL against methicillin-resistant *Staphylococcus aureus* (MRSA) and exhibits MIC 0.25 µg/mL against drug-resistant mycobacteria. Similarly, quercetin shows a MIC of 2 mg/mL against oral pathogens, such as *Streptococcus mutans* and *Lactobacillus acidophilus*. Furthermore, endophytic fungi isolated from *N. sativa* have demonstrated antibacterial activity. Therefore, *N. sativa* is a valuable medicinal plant with potential for medicinal and food-related applications. In-depth exploration of the corresponding therapeutic potential and scope of industrial application warrants further research.

## 1. Introduction

The unselective and inappropriate use of antibiotics in human healthcare has led to the rapid emergence and propagation of antibiotic resistance in pathogenic bacteria, whereby these microbes acquire resistance to a wide range of common antibiotics, a phenomenon known as multidrug resistance (MDR) [1]. Antibiotic-resistant pathogens pose a great challenge in the global healthcare sector, as they increase morbidity, mortality, the economic burden associated with infectious disease incidence, and the need to find effective alternative treatments against them (Centers for Disease Control and Prevention, 2019) [2]. Indeed, the increasing number of MDR microbial strains identified in community-acquired infections has become the cause of increasing global alarm [3]. In this situation, the World Health Organization (WHO) prioritizes the development of alternative drugs, considering the alarming increase in the number of MDR species and their harmful effects on the health of people worldwide [4]. Therefore, scientists, clinicians, and researchers are working to test different agents as substitutes for antibiotics, such as bacteriophages, bacteriocins, nanoparticles, and seed and herbal extracts with antimicrobial properties [5]. Besides the usefulness of plant-based antimicrobial agents against infections, there is a potential chance of resistance development in pathogens against them, too, particularly in case of prolonged use. This possibility can be predicted by the extensive research reporting the emergence of antimicrobial resistance in pathogens owing to the prolonged or repeated use of conventional antibiotic drugs [6]. Several studies have reported the detrimental effects of antibiotic drugs on gut microbiota composition and gastrointestinal health, raising concerns that long-term use of phytochemical drugs for infectious diseases in clinical settings could lead to similar issues [7]. However, medicinal plants, such as *N. sativa* (commonly known as black seed or cumin), with their rich array of bioactive compounds, offer potential therapeutic benefits that could help mitigate such health problems. The species is predominantly found in Uzbekistan, Egypt, Southern Europe, Southwest Asia, North Africa, India, and Pakistan. In South Asia, it is called “Kalongi”, and its Arabic name is “Habib-ul-Saua”. It is very important in Greek medicine and Islamic culture, with a long history of conventional use, and is well known for its broad-spectrum pharmacological characteristics [8,9]. Cumin essential oil yields obtained in different countries are listed in Table 1. Most Nigella seeds contain over 30% oil, with some reaching up to 40% [10]. Yield variations arise from environmental factors, such as water stress, saline conditions, and cooler temperatures, reducing yields to 13–23% in places such as Italy [11], similar to regions such as Northwestern Morocco. Both genetic differences and environmental factors, such as altitude and temperature, significantly impact yields and bioactive compound levels, as seen in Indonesia, Kuwait, and India [12]. The method of extraction also affects yield, with solvent extraction being more efficient than cold pressing [13]. These variations are crucial in determining the medicinal and nutritional quality of the oil.

**Figure 1:**
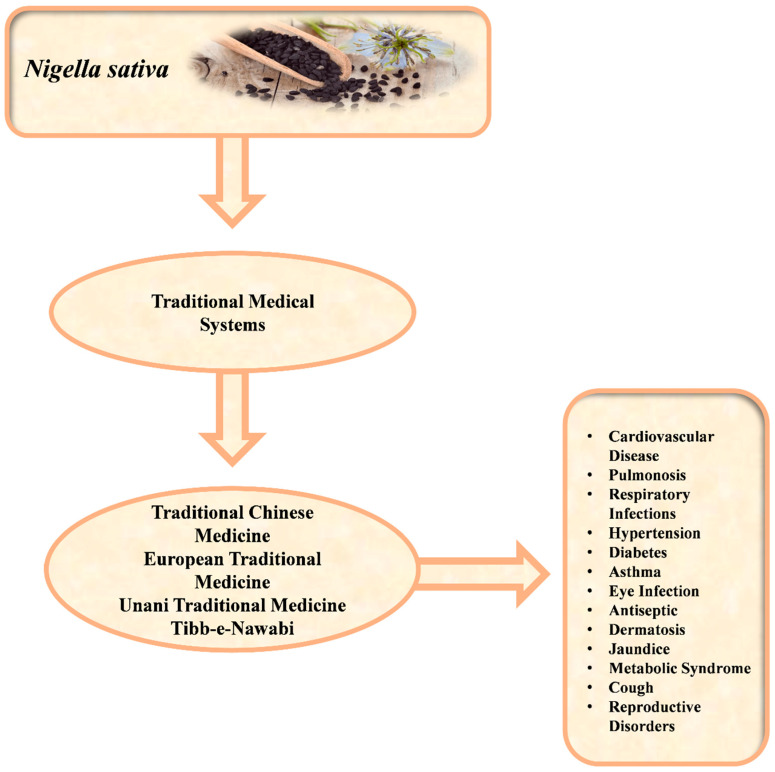
*Nigella sativa* used in different traditional medical systems for the treatment of various diseases.

Natural compounds with in vitro therapeutic potential are abundant in *N. sativa*, and include organic antioxidants with antidiabetic, anti-inflammatory, anti-cancer, and wound-healing properties, as well as antibacterial and antifungal activities. *N. sativa* has long been used traditionally for treating bacterial infections, such as in Iran and Pakistan [25,26]. Empirical studies have now confirmed its antibacterial properties, with modern research showing thymoquinone’s efficacy against drug-resistant strains, such as methicillin-resistant *Staphylococcus aureus* (MRSA) and mycobacteria [27,28]. Historically, cumin has been used to treat various illnesses worldwide, especially in Eastern Europe, the Middle East, Western Asia, and Central Asia [29] (Figure 1). Its importance in the Islamic culture derives from it being considered a curative drug [30]. Cumin seeds’ physical and nutritional properties are well known [31]. Although the volatile oil from *N. sativa* seeds has neuroprotective and therapeutic effects against sciatic nerve injury, these effects have mainly been observed in terms of neuronal number and morphology [32]. Overall, cumin seed oil can be considered a potential food supplement to enhance memory, attention, and cognition [33].

*Nigella sativa* seeds are rich in alkaloids, proteins, saponins, and fixed and volatile essential oils. Particularly, thymoquinone (TQ), the main constituent of the essential oil, is responsible for a large portion of the biological activity of the seed extracts. The fixed oil contains mainly O-cymene [34]. Meanwhile, the lipid portion of black seed contains valuable fatty acids and sterols, such as linoleic and oleic acids, b-sitosterol, and stigma sterol. In turn, proteins and peptides isolated from *N. sativa* reportedly exhibit a variety of bioactive effects, such as antibacterial, anti-cancer, and anti-inflammatory [9]. All these compounds have shown moderate activity against *Staphylococcus aureus* but poor activity against *Shigella* species and *Klebsiella pneumoniae* [35]. Essential fatty acids, glycolipids, phospholipids, and bioactive phytosterols are abundant in the fixed oil [36]. Further, the antibacterial efficacy of the essential oil extracted from *N. sativa* seeds against clinical isolates of bacteria resistant to different antibiotics was investigated using the “disc agar diffusion technique” and saturated filter-paper discs on inoculated Muller Hinton agar plates. The results showed that Gram-positive bacteria were more susceptible than Gram-negative ones to the antibacterial action of the essential oil in a dose-dependent manner [37].

Cumin is one of the best-ranked, evidence-based herbal remedies and, by and large, TQ is the key component responsible for the medicinal properties reported [30], and with a long history of use in conventional medical systems, including Chinese, Greco-Arabic, Islamic, Unani, and Ayurvedic, *N. sativa* is an excellent source of TQ. In addition to the above-mentioned curative effects, TQ is a potential treatment for skin diseases because of its antimicrobial, anti-inflammatory, and antineoplastic properties [38].

Research has shown that the aqueous *N. sativa* extract does not exhibit any microbial activity against *Pasteurella multocida,* whereas the ethanolic extract shows some activity against this strain but none against *S. aureus* [39].

Additionally, with an oil content exceeding 35%, there is great interest in using cumin seeds as a “feedstock” for the production of biofuels. The transesterification reaction for this process can be initiated through alkali-, acid-, or enzyme-catalyzed methods. However, the efficacy of the primary catalysts may be significantly diminished when the feedstock contains substantial amounts of fatty acids, whose presence in cumin oil poses a challenge to the use of homogenous alkali catalysts, as they lead to saponification, thereby depleting the catalysts and increasing subsequent purification costs. Although enzymes can facilitate a straightforward purification process for biodiesel and glycerol, they may not be suitable for industrial-scale applications [40].

Additionally, it has been demonstrated that endophytic fungi isolated from *N. sativa* are a rich source of organic chemicals with biological activity, including bioactive metabolites derived from fermentation in PDA culture media. Thus, crude endophyte extracts have shown promising antibacterial activity against both Gram-positive and Gram-negative bacteria [41].

Lastly, high-quality antioxidant-rich *N. sativa* extracts can be produced via supercritical fluid extraction [42].

The objective of this review is to integrate the critical clinical aspects of *N. sativa* to facilitate the identification of more active ingredients and their significance to human health.

## 2. Bioactive Compounds Found in *N. sativa*

**Figure 2:**
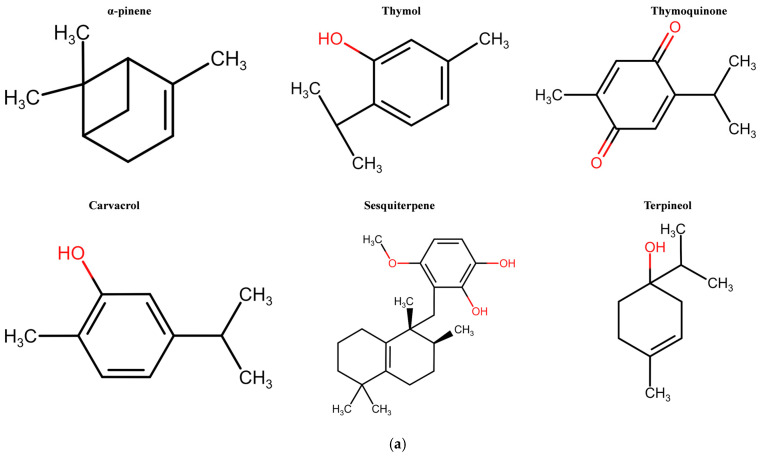

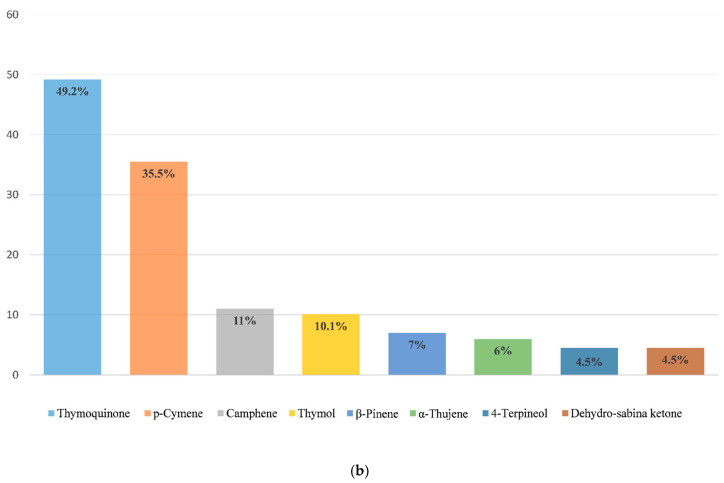
(**a**) Chemical structures of key bioactive compounds from *Nigella sativa*. (**b**) The graph illustrates the relative prevalence of thymoquinone, p-cymene, camphene, thymol, terpinol, and alpha-thujene, highlighting their significance among the wide range of bioactive constituents found in *Nigella sativa*. The compounds were quantified using different techniques, such as gas chromatography (GC) and mass spectrometry (MS), followed by different extraction methods, such as supercritical fluid and Soxhlet extraction.

Naturally occurring compounds can alter living cells. In particular, *N. sativa* seeds contain carbohydrates, proteins, lipids, and vitamins, and specific combinations and interactions of these macromolecules contribute to their crucial role in improving the immune system and its functionality [43]. Recently, several bioactive substances from a range of black cumin seed variants have been extracted, identified, and recorded. These compounds are distinguished by their specific biological activities and impacts on human health, which determine their therapeutic roles in various medical conditions [44]. *N. sativa* mainly contains alpha-hedrein, carvacrol, nigellimine, N-oxide, nigellicine, p-cymene, carvacrol, 4-terpineol, t-anethole, sesquiterpene, α-pinene, thymol, TQ, and alkaloids, such as pyrazole alkaloids and uncommon indazole ring alkaloids that show remarkable biological activities [45,46,47] (Figure 2a). Furthermore, the distribution of bioactive compounds in *N. sativa* has been comprehensively examined, with an emphasis on composition diversity [46] (Figure 2b).

Studies have shown that black cumin seed oil primarily consists of fatty acids, with linoleic and palmitic acids accounting for 64.6% and 20.4% of their composition, respectively. Further, the seed oil contains 0.4–2.5% essential oil [48,49]. The seeds contain a remarkable number of fibers, with soluble fibers comprising from 20.5 to 27.1 g 100 g^−1^, while the insoluble portion reaches up to 6.5–8.9 g 100 g^−1^. Moreover, 18–42% of black cumin seed oil is composed of sterols, predominantly β-sitosterol, campesterol, stigmasterol, and 5-avenasterol [17]. In turn, α-, β-, and γ-tocopherol add up to 9.15–27.92 mg 100 g^−1^ of the seed oil [50,51].

Black cumin seeds are a good source of saponins, including alpha-hederin, a water-soluble pentacyclic triterpene with potential anti-cancer effects. Additionally, studies have found trace quantities of some other compounds, such as carvone, limonene, citronellol, flavonoids, coumarins, and tannins [52,53,54]. The exact composition of the various elements found in cumin seed oil is determined by different factors, e.g., the specific plant source, the conditions under which the seed was stored, and the method of extraction used, such as supercritical CO_2_ extraction (SC-CO_2_; 1.06–4.07 mg g^−1^) or Soxhlet extraction (2940.43 mg kg^−1^ and 8.8 mg g^−1^) [55,56]. Furthermore, phytochemical analysis has revealed the presence of over 100 phytonutrients in *N. sativa* seeds, although the chemical characteristics and biological activities of a significant number of these nutrients remain unidentified and unverified. Below, we summarize our current knowledge on the specific biological functions and health benefits of various types of chemical compounds found in *N. sativa* seeds.

### 2.1. Terpenes and Terpenoids

#### 2.1.1. Thymoquinone

Generally known as TQ, 5-isopropyl-2-methyl-1,4-benzoquinone is the most important bioactive compound isolated from *N. sativa* seed oil. This compound has various therapeutic effects on the human body [57], including, in addition to those mentioned above, effects on metabolic disorders, such as obesity, diabetes mellitus, dyslipidemia, high blood pressure, and metabolic pathway disturbances, which lead to cardiovascular disease (CVD) [58]. Indeed, several studies have focused on the evaluation of the pharmaceutical significance of TQ and its derivatives [59,60]. Thus, many studies on humans and animals have verified the potential role of TQ in balancing the serum lipid profile [61]. Moreover, TQ can reduce the activity of HMG-CoA reductase in the liver, enhance erylesterase, and regulate genes involved in cholesterol metabolism, ultimately preventing dyslipidemia and other metabolic conditions [62]. Furthermore, various studies have reported that the role of TQ in cardiovascular disease treatment might be closely associated with its ability to reduce the activity of β-hydroxy β-methylglutaryl HMG-CoA reductase and, subsequently, lower total cholesterol levels. Experiments in rats have demonstrated that TQ effectively alleviates hypertension [63].

#### 2.1.2. P-Cymene

Chemically a monoterpene, p-cymene is a prominent component found in essential oils derived from diverse plant species [64]. Some studies revealed that p-cymene exhibited antioxidant properties in vivo, while holding potential as a neuroprotective agent within the brain. This compound could offer a novel approach to crafting treatments for various diseases wherein oxidative stress significantly contributes to the observed pathophysiology [65]. P-cymene has an extensive range of pharmacological effects, including antibacterial, antioxidant, anti-inflammatory, antiparasitic, antidiabetic, antiviral, and antitumor activities. Particularly, p-cymene has potential as an anti-inflammatory agent by effectively modulating cytokine production, including tumor necrosis factor-α (TNF-α), interleukin-1β (IL-1β), interleukin-6 (IL-6), and interleukin-10 (IL-10), through inhibition of nuclear factor-κB (NF-κB) and mitogen-activated protein kinase (MAPK) signaling pathways. Additionally, studies have indicated its analgesic/antinociceptive and immunomodulatory properties, along with the hypotensive and bradycardic effects observed in urethane-anesthetized rats [66]. Further, p-cymene serves as a significant industrial intermediate and is used in the manufacture of fungicides, pesticides, perfumes, and fragrances; lastly, it is also used in the production of certain precursors of common antioxidants, such as p-cresol [67].

#### 2.1.3. Carvacrol

Known as carvacrol, ‘5-isopropyl-2-methylphenol’ is an important monoterpene obtained from black cumin seeds with a range of biological activities, including antibacterial and antifungal properties [68]. Carvacrol inhibits the proliferation of both Gram-positive and Gram-negative bacteria, successfully deterring biofilm formation. It has emerged as a potential substitute for conventional antimicrobial agents against MDR bacteria. This bioactive compound is prevalent in various plants, such as oregano, thyme, sweet basil, black cumin, and food savory, and is used for medicinal purposes in many regions. In addition to its pharmacological applications, carvacrol is also used as a feed supplement to enhance the performance of livestock and improve the quality of meat and eggs [69]. Researchers continue to explore its bioactive properties and how to take advantage of such properties in the health and livestock industries. Despite their promising medical prospects, further investigations into the toxicity and potential side effects of these compounds are needed [70].

The supplementation of poultry feed with carvacrol has demonstrated efficacy in enhancing the quality of poultry meat by suppressing tissue lipid oxidation, a significant deterioration process that affects both the sensory and nutritional attributes of food. This feed supplementation approach offers a straightforward and convenient method for introducing lipid-soluble antioxidants into phospholipid membrane tissues that can effectively prevent oxidative reactions at localized sites. Additionally, increasing concerns regarding the safety of synthetic antioxidants, such as butylated hydroxytoluene or butylated hydroxyanisole, have spurred further investigation of plant constituents, such as carvacrol [71].

#### 2.1.4. Camphene

Camphene is a monoterpene extracted from *N. sativa* seeds. A study showed that this compound altered cholesterol and triglyceride levels. The experiment involved normal rats and rats exposed to detergents that made them prone to high cholesterol levels. Camphene administration significantly reduced LDL cholesterol and triglyceride levels in rats with increased cholesterol levels. The mode of action of camphene differs from that of mevinolin and other cholesterol-lowering drugs. It lowered blood cholesterol levels without effecting HMG Co-A reductase. The same study demonstrated the anti-cancer activity of camphene, which was a result of apoptosis induction in melanoma cells via an intrinsic pathway, along with the release of calcium ions, HMG B1, and calreticulin, which cause stress in the endoplasmic reticulum. Furthermore, the importance of this compound has increased because of its antitumor activity in aggressively growing melanoma cells [72].

#### 2.1.5. Thymol

Thymol, another compound found in *N. sativa*, provides diverse health benefits. Research indicates that thymol, along with other constituents, such as thymoquinone, exhibits antimicrobial, anti-inflammatory, and COX-1 and COX-2 inhibitory properties, suggesting considerable potential as a nonsteroidal anti-inflammatory agent [73,74]. Studies have also focused on the potential of *N. sativa*-derived thymol as an anti-proliferative and anti-cancer agent, highlighting its ability to suppress cell proliferation and combat cancer [75]. *N. sativa* is associated with a broad spectrum of therapeutic effects, including antioxidant, anti-inflammatory, cough-suppressing, gastroprotective, anxiolytic, ulcer-preventing, asthma-reducing, cancer-fighting, immune-modulating, and liver-protective effects [76].

#### 2.1.6. Terpineol

Alpha-terpineol (α-T), a monoterpenoid present in numerous essential oils, has extensive applications in fragrances, cosmetics, culinary and domestic products, and antiseptic agents; moreover, it shows significant bioactivity [77], including antihypertensive, antioxidant, analgesic, gastroprotective, anticonvulsant, and sedative properties. Furthermore, α-T enhances transdermal penetration and has potential as a natural insecticidal agent. These characteristics render it a promising candidate for pharmaceutical and agrochemical applications [78]. Finally, terpineol, particularly 4-terpineol found in the essential oil of black cumin seeds, demonstrated significant radical-scavenging properties and effective antioxidant activity in various assays, including lipid peroxidation and deoxyribose degradation [79].

### 2.2. Alkaloids

#### 2.2.1. Nigellidine

Nigellidine is an alkaloid that belongs to a group of naturally occurring compounds containing basic nitrogen atoms [80]. It is a bioactive compound extracted from *N. sativa* seeds using column and thin-layer chromatography techniques [55]. The biological activity of nigellidine is attributed to its chemical structure, which contains an indazole ring system [81]. Previous studies have suggested that nigellidine exerts various pharmacological effects. One of these studies indicated that nigellidine may substantially inhibit TNF-induced inflammatory signaling and Fas-induced apoptotic death signaling, demonstrating greater efficacy than the positive control drug, oseltamivir [82]. Nigellidine from *N. sativa* showed strong potential in targeting COVID-19 proteins and inhibiting IL1R–IL6R, demonstrating antioxidative, hepato-reno-protective, immunomodulatory, and anti-inflammatory activities [83].

Several chemical variants of nigellidine have been isolated from black cumin as well. Nigellidine-4-O-sulfite was the first sulfated alkaloid of this type. Nigellimine and nigellimine N-oxides are isoquinoline alkaloids. Several dolabellane-type diterpene alkaloids, nigellamines A1–A5, have been isolated from *N. sativa*. Natural indazole-type alkaloids were obtained only from *N. sativa*, indicating that they might serve as potential taxonomic markers [84].

#### 2.2.2. Nigellamines

Black cumin seeds are rich in nigellamines, particularly nigellamines A1 and A5, which are diterpene alkaloids found in *N. sativa* seeds that are known for their significant biological activities. An experimental study demonstrated that nigellamines A3, A4, A5, and nigellamine C, obtained from the essential oil of black cumin seeds, are capable of lowering triglyceride levels in mouse hepatocytes; specifically, the effect of nigellamine A5 is comparable to clofibrate [85]. Moreover, the synthesis of other nigellamine alkaloids is now possible, as scientists have synthesized nigellamine A2 and confirmed its stereochemical confirmation [86]. Other *Nigella* species also contain a wide range of alkaloids, such as a novel compound called nigeglanine, which comprises an indazole nucleus isolated from *N. glandulifera* seeds and has pharmacological properties. A previous study showed that nigeglanine had a positive impact on intestinal health by protecting the colon from loss of length and preventing epithelial cell damage [87]. Additionally, several studies have demonstrated the therapeutic strength of nigellamines, mainly A1 and A5, in regulating lipid metabolism [88].

### 2.3. Polyphenols

#### 2.3.1. Vanillic Acid

Roots and shoots of *N. sativa* plants are good sources of polyphenols, such as vanillic acid (VA), obtained in the methanolic extract [88], which shows neuroprotective activity and is effective in the management of vascular dementia and cerebrovascular failure. Its pharmacological significance is due to its role in mitigating inflammation and the symptoms of various neurological diseases [89]. Owing to its anti-inflammatory characteristics, this polyphenol plays a therapeutic role in asthma and other respiratory disturbances, as it reduces pro-inflammatory cytokines, subsequently relieving inflammation in the lungs and air passageways [90]. Numerous studies have reported that VA can be used as a constituent of functional foods and as a dietary supplement for the prevention of neural disorders [91].

#### 2.3.2. Caffeic Acid

Also known as caffeic acid (CA), 3,4-dihydroxycinnamic acid is a polyphenolic compound with strong antioxidant characteristics found in black cumin seeds. Besides being a potent antioxidant, CA possess anti-inflammatory, immunoregulatory, and anti-cancer effects [92]. The chemical conformation of CA, characterized by free phenolic hydroxyls and a double bond in the carbonic chain, enables it to exert antioxidant and pro-oxidant activities in metastatic cells, making it a potential anti-cancer agent [93]. A previous study demonstrated that CA also stimulates apoptosis in multiple myeloma cells via the caspase-dependent pathway [92].

#### 2.3.3. Flavone

Flavone is another phenolic compound obtained in the methanolic extracts obtained from roots and shoots of the black cumin plant [88]. A recent study investigated the impact of a crude flavonoid extract derived from *N. sativa* and reported that it inhibited the proliferation of MCF-7 human breast cancer cells and is, therefore, a potential therapeutic agent for the treatment of breast cancer [94]. Another study that focused on *N. sativa* as a reducing agent found that an aqueous extract of black cumin seeds can be used as a coating for gold nanoparticles, as well as a reducing agent. Further experiments using the alpha-amylase method demonstrated considerable antidiabetic effects of these nanoparticles [95].

#### 2.3.4. Catechins

Nigella roots and shoots are a good source of catechins, which have significant anti-inflammatory and antioxidant properties, which make them important pharmacological agents [96]. Catechin can increase the production of adipocytes and prevent TNF-α-induced inflammatory responses [96].

### 2.4. Fatty Acids

*Nigella sativa* seeds of are rich in a wide range of saturated and unsaturated fatty acids, including palmitic, lenoleic, stearic, oleic, butyric, lauric, and myristic acids, which exhibit several features that can be useful in therapeutics [97].

#### 2.4.1. Palmitic Acid

Palmitic acid is a saturated fatty acid that accounts for 12.5% of the black cumin seed oil [98]. It is involved in numerous metabolic mechanisms, including modulation of CD-36 cells and AMP-activated protein kinase [99]. A study on the lipid content of *N. sativa* and its effects on the immune system reported that palmitic acid plays a direct role in increasing the secretion of interleukin 6 (IL-6) and 3T3-L1 adipocytes [100]. A previous study reported that intracerebroventricular (icv) injections of palmitic acid in experimental mice led to reduced leptin sensitivity and leptin-induced changes in liver gluconeogenesis and lipogenesis. These findings demonstrate the role of palmitic acid in the management of homeostatic disorders associated with the liver and obesity [101].

#### 2.4.2. Linoleic Acid

Linoleic acid is another important fatty acid found at the highest concentration (58.9%) in the essential oil of *N. sativa* and contributes significantly to its nutritional value [98,102]. Linoleic acid mitigates and prevents obesity-induced health issues [103]. Moreover, the cytotoxic and anti-cancer properties of *N. sativa* seeds have also been attributed to linoleic acid, as, in particular, it stimulates apoptosis in ovarian carcinoma and can, therefore, be utilized as an anti-cancer therapeutic agent [104].

#### 2.4.3. Oleic Acid

Oleic acid is a monosaturated fatty acid that accounts for 28% of the oil in black cumin seeds [98]. Oleic acid has several health benefits, including improvement of insulin sensitivity, reduction of inflammation, and prevention of insulin resistance [105]. Oleic acid enhances carnitine palmitoyltransferase 1 (CPT-1) levels and forces fatty acids into the mitochondria, thereby reducing ceramide in cells exposed to high palmitic acid [106]. Oleic acid was also found to have a protective effect on hepatocytes by inducing autophagy and triacylglycerol production, thereby preventing apoptosis of these cells [107]. It also plays a role in protecting the human body from atherosclerosis and insulin resistance associated with the cardiovascular system [105].

### 2.5. Phytosterols

The black cumin seed oil has a comprehensive chemical profile that also includes phytosterols, such as β-sitosterol, campesterol, stigmasterol, and 5-avenasterol, exhibiting numerous health advantages. These plant-based sterols assist in lowering the cholesterol levels in the body [108].

#### 2.5.1. Campesterol

Campesterol, an important bioactive compound present in *N. sativa* seeds, has also been studied for its health benefits. Research has shown that campesterol is a good therapeutic option for arthritis, as it reduces paw edema in rats and lowers the levels of inflammation-inducing cytokines, such as IL-1, TNF-α, NFκ-B, IL-6, and COX-II. Furthermore, campesterol stimulates the production of anti-inflammatory interleukin-4 (IL-4) and induces homeostasis of blood chemistry, subsequently alleviating the severity of symptoms in rheumatoid arthritis patients [109].

#### 2.5.2. Cholesterol

Plant-based lipids and cholesterol are good for health because they are mostly comprised of high-density lipoproteins (HDL), while animal sources increase the level of low-density lipoproteins (LDL), generally regarded as “bad cholesterol”. Black cumin seed oil intake reduces the risk of cardiovascular disorders by balancing serum cholesterol [110]. In addition, detailed studies have reported anti-hypercholesterolemic effects of *N. sativa*. Due to the presence of various phytosterols, the seed oil of *N. sativa* can boost arylesterase function, an indicator of cardiovascular health, while reducing serum HMG-CoA reductase levels, thereby helping in regulating the lipid profile [111,112].

#### 2.5.3. Stigmasterol

Stigmasterol is an unsaturated plant sterol, classified as a tetracyclic triterpene. Among phytosterols, stigmasterol is the most frequently extracted sterol from the oils of numerous plants, herbs, and vegetables, and has a wide range of therapeutic and pharmacological applications [113]. Stigmasterol is a food additive recognized as E499 in the European Union and is used in the food industry to increase phytosterol content and balance LDL cholesterol in various products [114]. It is a C24 alkylated cholesterol that functions as a component of the cell membrane and contributes to its strength and stability [115]. Stigmasterol increases the transport of glucose transporter type 4 (GLUT4), reduces fasting blood glucose levels, and treats insulin resistance; therefore, it is a potential antidiabetic agent [116]. This phytosterol has also been found to be therapeutic against skin, ovary, breast, prostate, and gastrointestinal tract carcinomas, contributing to the anti-cancer properties of *N. sativa* [117]. Moreover, studies have reported the role of stigmasterol as a strong antioxidant, anti-inflammatory, antimicrobial, immunoregulatory, and neuroprotective compound [118,119,120,121].

Several strains of viruses, fungi, and bacteria react differently to multiple bioactive substances, exhibiting distinct activities and modes of action, based on their different functions (Table 2).

## 3. In Silico Toxicity and Drug-Likeness Evaluation

To determine the potential of *N. sativa* compounds as therapeutic agents, a comprehensive overview of their toxicity profiles, cLogP, solubility, topological polar surface area (TPSA), drug-likeness, and drug score was evaluated using the Osiris Property Explorer (https://www.organic-chemistry.org/prog/peo/ accessed on 14 May 2024). Toxicity evaluation helps to understand the potential adverse effects that may arise from the consumption or application of these compounds. Their mutagenic, tumorigenic, and irritant properties were evaluated to gain valuable insights into their potential therapeutic value. For example, nigelidine, carvacrol, longifolene, p-cymene, and nigellimine have no mutagenic, tumorigenic, and irritating effects, indicating their potential for therapeutic use. Conversely, compounds such as lauric acid, camphor, carvone, benzaldehyde, and coumarin do have mutagenic and tumorigenic properties, whereby their therapeutic use advises caution (Table 3).

However, compounds such as nigellidine and epicatechin show favorable drug-likeness and high drug scores, suggesting their suitability for drug development. Conversely, compounds such as pentyl hexadec-12-enoate and lauric acid show poor drug-likeness and low drug scores, indicating their limited potential as viable drug candidates (Table 4). Based on this detailed toxicity assessment, the safety profiles and pharmacokinetic properties of the bioactive compounds present in *N. sativa* were evaluated to ensure their efficacy and safety in clinical trials. These properties provide researchers and clinicians with a better understanding of the therapeutic potential of *N. sativa* that will facilitate the identification and development of safe and effective antimicrobial agents without adverse effects.

## 4. Antioxidant and Immunomodulatory Properties of *N. sativa*

**Figure 3:**
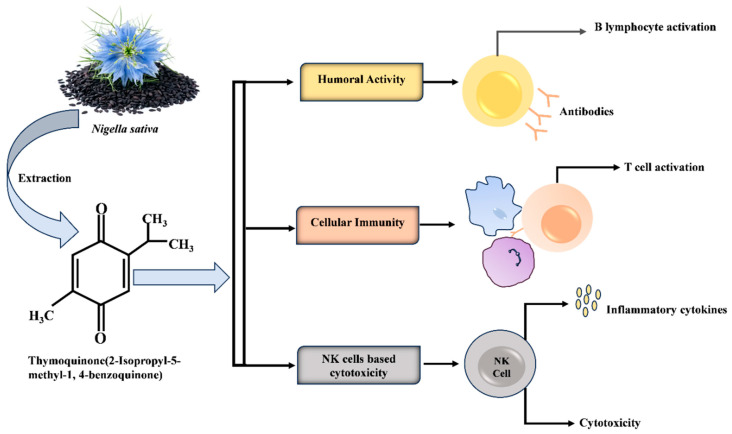
Schematic illustration of the proposed immunomodulatory pathways of thymoquinone. Thymoquinone stimulates B and T lymphocyte activation, promotes antibody production, regulates the release of cytokines (TNF, IL-1, and IL-6), and increases the cytotoxicity of NK cells.

Antioxidants are molecules that inhibit the action of free radicals in the body and are highly damaging to health. Chemical analysis of the essential oil and various extracts of *N. sativa* have revealed their antioxidant properties [139]. By enhancing the activity of antioxidant enzymes, such as glutathione peroxidase, glutathione reductase, glutathione S-transferase, and catalase, TQ aids in neutralizing free radicals and reactive oxygen species (ROS) [139,140]. Furthermore, cyclooxygenase and 5-lipoxygenase in the arachidonic acid metabolic pathway are blocked by various bioactive chemicals found in *Nigella* seed oil. Thin-layer chromatography was used to separate TQ, carvacrol, t-anethole, and 4-terpineol from the black cumin seed oil. Additionally, their synergistic antioxidant properties were confirmed using the 2,2-diphenyl-1-picrylhydrazyl (DPPH) test [141]. Clinical investigations have reported that Nigella seed oil, specifically TQ, reduces the arthritis activity scale score (disease activity score, DAS 28) and bone resorption in patients with rheumatoid arthritis. Similarly, another experimental study reported the excellent ability of *N. sativa* to affect inflammatory processes and reduce oxidative stress. Its components inhibit the activity of NF-κB by inducing IL-6, TNF-α, and other cytokines, consequently reducing inflammation [141]. Additionally, TQ has an inhibitory effect on eicosanoids and other pro-inflammatory factors of the immune system, including NF-κB/STAT3. NF-κB has been found to primarily induce inflammation, specifically in rheumatoid arthritis patients [142]. Further, researchers have studied the correlation between various extracts derived from *N. sativa* seeds and the in vitro response of peripheral blood mononuclear cells (PBMC) to different mitosis-inducing antigens (mitogens) [50]. Another in vitro study using splenic mixed lymphocyte culture and ethyl acetate column chromatography techniques proposed a considerable suppressive role of black cumin seed extracts in the humoral immune response [141]. In addition, *N. sativa* contains compounds capable of activating CD4+ T-helper cells that further differentiate into Th1 or Th2 cells, thereby affecting cytokine production and specific outcomes of adaptive immunity in various disorders. Reliable in vivo studies have also reported the immunoregulatory role of *N. sativa* in the T-helper-to-T-suppressor cell ratio and the cytotoxic effects of natural killer cells [143,144] (Figure 3).

## 5. Antimicrobial Activities of *N. sativa*

**Figure 4:**
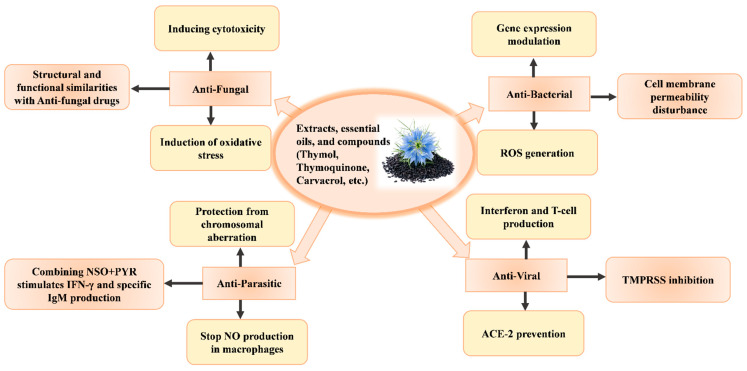
*Nigella sativa* exhibits protective effects against bacteria, fungi, parasites, and viruses through diverse and potent defense mechanisms.

The quest for adequate substitutes for existing antibiotic drugs with different modes of action against pathogenic microbes has led scientists to identify medicinal plants with potential antimicrobial capabilities [145]. Thus, numerous studies have shown that various plant phytochemicals, including anthraquinones, phenolics, flavonoids, alkaloids, terpenoids, saponins, and tannins, are beneficial against microbial infections. By preventing growth, altering the permeability of cellular membranes, interfering with metabolism, and modifying gene expression, these substances can kill bacteria, viruses, or fungal cells [146] (Figure 4). Studies investigating the antimicrobial strength of *Nigella sativa* have employed rigorous extraction techniques, clinical procedures, and statistical analyses to demonstrate its effectiveness against various pathogens. Shafodino’s study on the phytochemical profile and antimicrobial properties of black cumin seeds used the sequential maceration method for extraction, disk diffusion for antimicrobial testing, and SPSS version 24, with a significance threshold of *p* < 0.05 for statistical analysis [147]. Furthermore, the statistics of the antibacterial strength of *N. sativa* against different classes of bacteria showed that it is more effective against Gram-positive strains than Gram-negative ones (*p* < 0.0001) [148]. Among other species, *E. coli*, *P. aeruginosa,* and *S. aureus* were found to be more susceptible to the essential oil of *N. sativa* [147]. A subsequent study clearly highlighted the tannin content and antibacterial ability of two different varieties of *N. sativa* by using one-way ANOVA (Tukey’s test) [149].

### 5.1. Antibacterial Abilities

*Nigella sativa* seed extracts were studied for their antibacterial properties against various pathogenic bacterial species by adding varying concentrations of TQ to the bacterial culture. Subsequent measurement of minimum inhibitory concentrations (MIC) showed the antimicrobial potential of these extracts against *Pseudomonas aeruginosa* (ATCC 14886), *Bacillus subtilis* (ATCC 6633), *Escherichia coli* (ATCC 25922), and *Staphylococcus aureus* (ATCC 9144) [150]. Moreover, experiments have shown an effective inhibitory response of Nigella seed extracts against some strains of *Enterococcus faecalis*, *Aacinetobacterjunii*, *E. coli*, *S. aureus*, *Enterobacter cloacae*, *Serratia marcescens,* and *Proteus mirabilis*. Particularly, *E. faecalis*, *Acinetobacter junii*, *E. coli*, *P. mirabilis*, and *S. marcescens* were susceptible to the ethanolic extract, with a minimum inhibitory concentration of 5 mg/mL. Meanwhile, the methanolic extract had a 25 mg/mL MIC against *S. aureus*, whereas the isolates were resistant to the ethanolic extract. In the same experiment, MIC values of other extracts, such as chloroform, n-hexane, diethyl ether, butanolic, and acetonic extracts, against the above-mentioned pathogenic species demonstrated the effectiveness of *N. sativa* as an antimicrobial agent [151]. Another study focused the role of TQ in inhibiting biofilm formation using the crystal violet assay, colony-forming unit method, and scanning electron microscopy (SEM). TQ effectively degraded biofilms of *S. aureus*, *P. aeruginosa*, *E. coli*, and *B. subtilis* by producing ROS [150].

In light of the decreased effectiveness of existing antibiotics against bacteria, some studies have evaluated their use in combination with *N. sativa* to boost their antimicrobial activity and deal with antibiotic resistance. An experimental study reported the decrease in the MIC of several antibiotics when combined with oil extracts of *N. sativa* against resistant strains [152]. The collective use of β-lactam antibiotics and antibacterial compounds extracted from *N. sativa* was found effective against MRSA, causing greater bacterial inhibition and disruption of the bacterial cell wall [153]. Moreover, silver nanoparticles (AgNPs) obtained from *N. sativa* seeds can be integrated with streptomycin and ciprofloxacin to improve their antibacterial efficacy against MDR strains [154].

#### Activity of *N. sativa* Against *Mycobacterium tuberculosis*

Tuberculosis (TB) is a fatal respiratory infection caused by *Mycobacterium tuberculosis* (MTB), which affects over a million people every year globally [155]. According to the WHO, approximately three million people die of TB, whereas eight million silently develop various chronic infections, including TB. The administration of a combination of antibiotics for an extended period is a common practice for treating TB. However, these drugs have many side effects in patients, and their excessive use reduces bacterial susceptibility. The increasing number of MDR mycobacteria is a central issue for disease management purposes. Therefore, there is a need to replace antibiotics that have been in use for the past 40 years [156]. In view of this challenge, new therapeutic options are required to treat TB rapidly and effectively [157]. Several studies have been conducted in recent years to investigate the anti-mycobacterial potential of various extracts of *N. sativa*. Detailed research has shown that the MTB H37Rv strain is susceptible to the methanolic extract of *N. sativa*. In turn, the aqueous extract inhibited all three MDR strains of MTB; however, they were resistant to chloroform extracts [73]. Moreover, it has been shown to exhibit inhibitory action against TB at a concentration of 20 mg/mL [158]. Considering the extensive range of antimicrobial properties shown by *N. sativa* and the emerging demand for new remedies to combat mycobacterial infections, a clinical investigation demonstrated the in vitro activity of TQ against MTB isolated from the sputum specimens of patients with TB. Effective inhibition of MTB at a relatively low concentration of TQ, with a MIC of 20 μg/mL, was confirmed using the manual mycobacterial growth indicator tube method [158].

**Figure 5:**
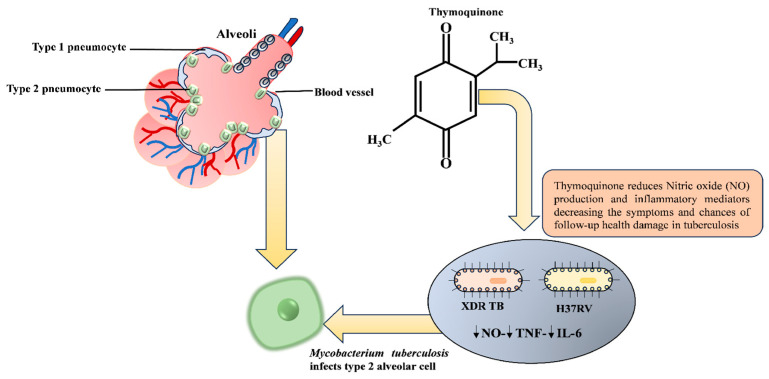
The schematic illustration demonstrates how thymoquinone reduces nitric oxide (NO) and inflammatory mediators, potentially alleviating symptoms and decreasing the likelihood of additional health complications in tuberculosis (TB). Additionally, the image emphasizes targeting type 2 alveolar cells by mycobacterium tuberculosis (MTB), underscoring the promising potential of thymoquinone in combating this infectious respiratory disease.

Bioactive substances derived from many plant sources, including 1,8-cineole, α-verbenol, citral b, TQ, piperitone, alantolactone, octyl acetate, and α-pinene, are under study for the development of novel medications against TB. These compounds are highly capable of disrupting the permeability of mycobacterial membranes [159]. Particularly, TQ derived from black cumin seeds has been reported to influence the mycobacteria-induced production of nitric oxide (NO) and inflammatory reactions in TB-infected type II human alveolar and myeloid cell lines, subsequently alleviating symptoms in patients with TB [160] (Figure 5). Indeed, TQ is efficient in immune regulation and exerts bacteriostatic action against intracellular pathogens. Importantly, this magic compound attacks drug-sensitive and drug-resistant strains of mycobacteria, with a lowest MIC of 0.25 µg/mL [161]. In addition to its antimicrobial activities, reports have found that TQ prevents possible hepatic dysfunction during anti-TB drug treatment, ensuring smooth and liver-friendly therapy [162].

### 5.2. Antiviral Activity

Bioactive components found in black cumin seeds also possess strong antiviral potential. These properties were studied extensively during the COVID-19 pandemic. Examination of the impact of Nigella seed oil on individuals with mild COVID-19 symptoms revealed that when patients were administered this oil at a dose of 500 mg/kg, there was a notorious attenuation of the symptoms of COVID-19. The group administered Nigella seed oil showed symptom alleviation for an average of approximately 10.7 days, compared to the 12.4-day average of the control group [163]. Several computational (in silico) studies have been conducted to better understand the antiviral activity against severe acute respiratory syndrome coronavirus 2 (SARS-CoV-2). An in silico analysis proposed that TQ can inhibit the activity of SARS-CoV-2 protease [164]. TQ is also involved in inducing viral oxidation, thereby protecting cells by modulating endosomes [165]. Additionally, molecular docking studies have found a possible anti-covalent role for TQ, which hinders viral protein interactions by preventing angiotensin-converting enzyme 2 (ACE2) [162]. Researchers have examined various natural compounds using computer simulation studies to detect their effects on the enzyme transmembrane serine protease 2 (TMPRSS2) [166]. They found that carvacrol (a bioactive compound in *N. sativa*) theoretically performed better at inhibiting TMPRSS2 than the control Comstat [166]. In contrast, thymol showed low inhibitory activity. Another computational study suggested that nigellone, also known as dithymoquinone, might block all essential targets of the coronavirus [167]. In addition, two compounds found in the seeds, nigellidine and kaempferol, are strongly associated with COVID-19 C19MP proteases [168]. However, the effectiveness of in silico approaches has limitations, including the need for high-performance systems to screen large compound libraries, reliance on structural data, and the requirement for validation through experimental and clinical studies, which may not be available for emerging targets [169,170]. Further randomized clinical trials are necessary to confirm the therapeutic potential of *N. sativa* in COVID-19 treatment.

Biologically active substances, particularly TQ, can be used as potential therapeutic agents against several viral infections, including influenza, dengue, Ebola, and hepatitis. The effects of *N. sativa* seeds on the pathophysiology and immunological response to H9N2 avian influenza virus (AIV) were investigated in Turkey [171]. The seeds of *N. sativa* boosted the production of T-helper and cytotoxic T cells, enhanced the expression of interferons, and reduced inflammatory mediators, consequently helping combat influenza viruses and other viral diseases [172].

Further, *N. sativa* compounds have been shown to be beneficial against dengue fever, a mosquito-borne global health risk. The extraction of secondary metabolites with antiviral characteristics from medicinal plants is a novel strategy owing to their distinctive structural and biochemical characteristics [173,174]. Lastly, TQ has been studied as an immune booster, as it significantly enhances antibody production and defends the body against various bacterial and viral infections [173].

### 5.3. Antifungal Activity of N. sativa

In addition to other antimicrobial properties, black cumin, a versatile healer, has a remarkable ability to fight fungal infections [175]. Researchers have conducted numerous studies to assess the antifungal activity of *N. sativa* against various fungal pathogens. The chemical composition of *N. sativa* includes a diverse range of bioactive compounds, each contributing to its unique biological properties. These compounds confer antifungal, cytotoxic, and other therapeutic effects, particularly TQ, which possesses substantial antifungal efficacy against pathogenic strains, such as *M. gypseum*. *Candida albicans*, *Aspergillus fumigatus*, *Cryptococcus neoformans*, *T. mentagrophytes*, *M. canis*, *M. gypseum*, *P. digitatum*, and *C. gloeosporioides* [139,176,177,178]. Moreover, nigellothionins extracted from black cumin seeds exhibit robust antifungal and cytotoxic properties [179]. *N. sativa* seed extract also has fungicidal activity against *Aspergillus flavus* and *Aspergillus parasiticus* [180].

Recent studies have confirmed the efficacy of black cumin against dermatophytes, such as *Candida* spp., *Aspergillus*, and *Alternaria*. The antifungal activity of methanolic extracts is superior to that of conventional treatments, such as clotrimazole. The concentration-dependent effects of black cumin ethanolic extracts suggest tailored therapeutic applications. The rich chemical composition of black cumin, which includes TQ, paves the way for novel antifungal interventions [181]. A previous study demonstrated the inhibitory effects of *N. sativa* seed oil on *Fusarium solani*. The hexane and ethanol fractions exhibited the greatest inhibitory effects (24–28 mm), surpassing those of the chloroform and methanol fractions (30–37 mm). Particularly, ethanolic extracts potently inhibit various fungi. The defense peptides (Ns-D1 and Ns-D2) from *N. sativa* seeds showed potent antifungal activity. This study highlighted the correlation between antimicrobial effects and components, such as thymohydroquinone, TQ, p-cymene, and longifolene, as well as the antimicrobial properties of unsaturated fatty acids and phenolics in black seed oil [182]. In 2013, Akansha et al. determined that the essential oils of clove buds and black cumin seeds, along with several other important components, exhibited antifungal properties against *P. digitatum* and *C. gloeosporioides*. The Poison food technique and MIC determination revealed significant growth inhibition of the pathogenic fungal strains. The synergistic effects of the combined essential oils and compounds were evaluated using fractional inhibitory concentrations (FIC), following CLSI guidelines [178]. Antifungal susceptibility testing (AFST) was conducted on 40 *C. glabrata* isolates using CLSI standards (https://clsi.org/standards/, accessed on 15 May 2024). The data revealed that 72.5% of these isolates were resistant to fluconazole (FLZ), 12.5% to itraconazole (ITZ), and only 5% to amphotericin B (AMB), while TQ had an MIC50 of 50 µg/mL. Biofilm formation in *C. glabrata* was reduced two-fold when exposed to the MIC50 of TQ, compared to that recorded for the control group, as assessed by an MTT assay [183].

### 5.4. Antiparasitic Features

The distinct chemical composition and structural characteristics of *N. sativa* seed oil bioactive components account for the wide range of antibacterial properties observed upon its application. The immunomodulatory, antioxidant, and cytotoxic effects of *N. sativa* have been demonstrated for the management of parasitic disorders and other infectious diseases.

Comprehensive research has demonstrated the antimalarial activity of ethanolic, aqueous, and chloroform extracts upon intraperitoneal administration in varying quantities. The initial assessment of *N. sativa* seed extracts revealed that all three extracts exhibited inhibitory effects against *P. berghei* infection in mice. Interestingly, higher doses did not necessarily result in greater suppression, except for the aqueous extracts used at 400 µL/kg, which showed significant suppression. The inhibitory effect of *N. sativa* oil and its components is due to its antioxidant properties, which prevent NO production in macrophages, a potent intracellular mechanism for killing parasites. This process also leads to the upregulation of secondary immune mechanisms, which the parasite cannot effectively counteract, ultimately weakening its defense and contributing to its clearance by the host’s immune system [184]. *N. sativa* seeds can render parasites susceptible to host damage, particularly schistosomiasis. Furthermore, *N. sativa* significantly protected cells against chromosomal aberrations induced by *Schistosoma mansoni* infection. In addition, it may protect against schistosomiasis by modulating the immune response and reducing inflammation [139].

Protozoan parasite-induced diseases cause considerable morbidity and affect the well-being of more than 500 million people worldwide. The pursuit of novel anti-protozoan medicines has escalated, particularly that of botanical remedies. TQ has demonstrated remarkable effectiveness against *Entamoeba histolytica* and *Giardia lamblia*, surpassing the potency of the commonly used drug metronidazole [185].

A previous study examined the effectiveness of *N. sativa* oil (NSO), applied either by itself or in conjunction with pyrimethamine (PYR), for the treatment of *Toxoplasma gondii*-caused toxoplasmosis. This research was conducted in a cohort of 100 albino mice, and the therapeutic interventions were compared with the prior amalgamation of clindamycin (CLN) and PYR. The study employed the virulent RH Toxoplasma strain to assess survival rates, liver and spleen impression smears, and histopathological and ultrastructural analyses. The evaluation also included measuring the levels of interferon-γ, total antioxidant capacity (TAC), specific IgM, and liver malondialdehyde (MDA) contents. The results revealed that NSO alone did not exhibit anti-Toxoplasma activity; however, when combined with PYR, it showed an efficacy similar to that of the CLN+PYR combination treatment, which enhanced survival rates, reduced parasite density, and alleviated pathological damage to the liver and spleen [186].

## 6. Pharmacological Applications of *N. sativa*

*Nigella sativa* seeds are rich in diverse compounds that exert metabolic effects on the human body. These seeds comprise a wide range of terpenes and terpenoids, including longipinene, longifolene, transcarbophyllene, β-caryophyllene, zonarene, and many others, each contributing to its overall pharmacological potential [147].

Investigations of the chemical profile of *N. sativa* for medicinal use have reported that TQ is the most multifaceted therapeutic compound. Phytosterols, such as stigmasterol, campesterol, cholesterol, β-sitosterol, and Δ5-avenasterol, enhance the complexity of the *N. sativa* chemical composition. Alkaloids, namely, nigellicine, nigellicimine, and nigellamines A1–A5, further enrich the biochemical diversity of plants. Tocols, including alpha-tocopherol, beta-tocopherol, and gamma-tocopherol, contribute to the antioxidant potential of *N. sativa*. The polyphenolic composition, which includes quercitrin, kaempferol, caftaric acid, and a spectrum of other compounds, contributes to the overall health-promoting attributes of the plant. This intricate blend of bioactive compounds in *N. sativa* highlights its pharmacological potential and warrants thorough investigation in the context of natural product pharmacology. A detailed understanding of these compounds provides a solid foundation for further research exploring the application of plants in various therapeutic domains [88,187,188,189]. The pharmacological characteristics of black cumin seed extract, specifically its major bioactive component, TQ, have been documented to exhibit a wide array of effects, including anti-inflammatory, antioxidant, antimicrobial, immunomodulatory, neuroprotective, cardioprotective, and anti-cancer activities [190].

### 6.1. Anti-Inflammatory Drug

*Nigella sativa* oil and TQ exhibit potent anti-inflammatory properties, as demonstrated in various disease models, such as encephalomyelitis, colitis, peritonitis, edema, and arthritis. These effects are attributed to suppression of “inflammatory mediators”, such as prostaglandins and leukotrienes [191], and in a study involving rats, oral administration of 4 mL/kg/day of *N. sativa* oil for 31 days resulted in reduced production of IL-4 and NO [192]. Administration of TQ at 10 mg/kg showed anti-inflammatory activity by inhibiting cyclooxygenase (COX) and 5-lipooxygenase (5-LPO) pathways in rats [193,194]. Another study found that a 5 mg/kg dose of TQ in rats lowered TNF-α and IL-1β levels in arthritis. The anti-inflammatory activity of *N. sativa* has been extended to humans, as evidenced by its use in geriatric patients with osteoarthritis [195].

### 6.2. Strong Antioxidant

An aqueous extract (200–250 mL) administered daily for five days in a clinical study with healthy subjects led to a significant increase in erythrocyte glutathione (GSH), a non-significant increase in superoxide dismutase (SOD), and a non-significant decrease in malondialdehyde (MDA) on the sixth day. The antioxidant activity was tested in vivo [196]. Supplementation of experimental rats with *N. sativa* oil or TQ increased ceruloplasmin levels, an extracellular antioxidant responsible for reducing Fe^2+^ to Fe^3+^. Both NSO and TQ showed a preventive effect against radiation in renal tissues, leading to an increase in paraoxonase and a reduction in hydroperoxide lipids. *N. sativa* seeds reduce the total antioxidant condition and oxidative stress index in irradiated rats at the renal level [197]. Treatment of rats with black cumin oil showed antioxidant activity characterized by reduced MDA and oxidized glutathione (GSSG) levels, and an increased hydrogen donor capacity [198].

### 6.3. Immunoregulatory Agent

Gholamnezhad et al. studied the immunomodulatory effects of *N. sativa* in Wistar rats. Wistar rats injected with 10% phytohemagglutinin (PHA), *N. sativa* supplementation at 50 g/kg improved animal weight and enlarged the spleen, responsible for particle clearance. The antioxidant effect of *N. sativa* was linked to its immune-stimulant activity, as evidenced by increased levels of IL-12, stimulating the production of TNF-α, IF-γ, and CD8 in the spleen of treated rats [199].

In another experiment, mice treated with cyclophosphamide (CTX) were administered *N. sativa* polysaccharides (NSSP) to induce immunosuppression. This treatment protected the thymus and spleen against CTX-induced damage with an increase in lactate dehydrogenase and acid phosphatase levels, indicating immunomodulatory effects. CTX administration led to reduced antioxidant capacity, SOD and CAT activities, and increased MDA content, all of which were mitigated by NSSP administration. High-dose NSSP significantly increased IL-2, IL-4, and IL-6 levels in mouse serum, decreased TNF-α levels, and regulated cytokine levels, demonstrating immunomodulatory effects. NSSP administration also upregulated PI3K protein expression, downregulated PTEN expression, activated the PI3K/Akt signaling pathway, inhibited TLR4/NF-B expression, reduced ROS and TNF-α levels, and exerted immunomodulatory effects [200].

### 6.4. A Substitute for Antibiotics

Considering the pressing issue of AMR, the development of novel therapeutic agents that are effective against bacterial infections has become paramount. Numerous plants, seeds, and herbs have antibacterial properties and can be used as substitutes for antibiotics against MDR pathogens [201].

Scientists are exploring the applications of phytochemicals in food, medicine, and other industries owing to their vast nutritional and antimicrobial features. *N. sativa* contains a wealth of phytochemicals that can be used as alternatives to conventional antibiotic drugs. Significant antibacterial activity in the fractions of the methanolic extracts of *N. sativa* against a range of pathogens, including *Pseudomonas aeruginosa*, *Escherichia coli*, and *Staphylococcus aureus,* has been demonstrated. MDR bacteria, such as *Staphylococcus saprophyticus* and *S. epidermidis*, are also susceptible to this inhibitory effect [202]. Further, cold-pressed *N. sativa* oils showed a synergistic effect when tested with antibiotics on methicillin-resistant *S. aureus* (MRSA), enhancing the bactericidal effect, especially in combination with ‘Augmentin’. Specifically, scanning electron microscopy revealed membrane deformation in bacterial cells [203]. After *N. sativa* seed oils, the liquid/aqueous extract showed antibacterial activity against both Gram-positive (*Micrococcus luteus*, *S. aureus*, and *Bacillus subtilis*) and Gram-negative bacteria (*Agrobacterium tumefaciens*, *Salmonella setubal*, and *Enterobacter aerogenes*) [204]. In turn, the n-hexane extract exhibited promising antibacterial activity against *S. aureus*, MTCC, *S. aureus*, and *Salmonella typhi* [205]. TQ inhibited the growth of several bacterial strains and significantly inhibited biofilm formation. When used together with antibiotics, it showed a synergistic effect against both Gram-positive and Gram-negative bacteria [206]. TQ, in the methanolic extract, and NSO (extracted and commercial) showed better inhibition of *Bacillus subtilis* and *Bacillus licheniformis* [207].

With a minimum inhibitory concentration ranging between 0.25 and 1 µL/mL, the n-butanol extract from *N. sativa* seeds shows strong antibacterial activity against *P. aeruginosa*, *Klebsiella pneumoniae*, and *Acinetobacter baumannii*. However, it does not work against *S. aureus* or *E. coli*. The high content of terpenoids and fatty acids in the extract is presumed to be the cause of the antibacterial action [208].

### 6.5. Neuroprotective Medicine

Despite the research progress described above, a lack of research on *N. sativa* neuroprotective benefits still prevails. According to in vitro studies, pretreatment with *N. sativa* oil dramatically increases the vitality of neuronal cells [209]. Furthermore, in cultured cortical neurons, the methanolic extract of black cumin seeds can regulate the release of amino acid neurotransmitters, such as glutamate, glycine, aspartate, and gamma-aminobutyric acid (GABA). Additionally, it has strong effects as an analgesic and on the central nervous system (CNS) [210,211]. Several studies have identified *N. sativa* and TQ as neuroprotective agents [212].

### 6.6. Cardioprotective Agent

A cholesterol-rich diet, oxidative stress, and hypercholesterolemia can contribute to the development of atherosclerosis, a CVD characterized by reduced elasticity and hardening of the arterial walls, potentially leading to heart strokes. Elevated serum cholesterol, LDL, and triglyceride levels are the main contributing factors to this condition [213]. According to previous studies, *N. sativa* dramatically lowers the serum levels of triglycerides, LDL, and cholesterol, thus improving the lipid profile [214]. A study examining the impact of TQ intake on the blood lipid profile of rabbits fed a high-cholesterol diet showed a notable reduction in the levels of total cholesterol, LDL, triglycerides, and thiobarbituric-acid-reactive substances. Concomitantly, there was an improvement in the HDL cholesterol concentration [215]. *N. sativa* oil reduced the serum cholesterol and triglyceride levels in normal rats by 15.5% and 22%, respectively [216].

A promising result reported of a daily dosage of 1 g of *N. sativa* powder for two months in humans led to a notable drop in LDL cholesterol and triglyceride levels, as well as an increase in HDL cholesterol in hypercholesterolemic individuals [217]. Another study on hypercholesterolemic patients also associated *N. sativa* intake with decreased cholesterol levels, indicating the potential benefits of normalizing lipid profiles and preventing heart problems [44].

### 6.7. Application in Cancer Treatment

Numerous studies have indicated that cancer exposure risk can be reduced by using various vegetables and fruits, and *N. sativa* is one such ingredient showing promising anti-cancer activity [218]. A diet containing *N. sativa* and honey reportedly shows protective effects against lung, colon, and skin cancers. The anti-cancer effect of *N. sativa* was first revealed when an improvement in the activity of natural killer cells was observed in cancer patients receiving multimodality immunotherapy [219]. The seed extract of *N. sativa* showed cytotoxicity against multiple cancer cell lines, such as Lewis lung sarcoma (LL/2) [220]. Further, with an IC_50_ value of 43 µg/mL, *N. sativa* oil demonstrated a substantial inhibitory impact against the human lung cancer cell line A-549 [220]. Recently, Al Sheddi et al. showed that *N. sativa* seed extract and oil significantly reduced the viability of human lung cancer cells and changed the shape of A-549 cells in a concentration-dependent manner [221]. In a study examining the effect of aqueous and alcoholic extracts of *N. sativa* on MCF-7 cells, the findings demonstrated the efficacy of black cumin extracts in deactivating and inactivating MCF-7 cell lines [222]. To evaluate the preventive effects of *N. sativa* oil against colon cancer, abnormal crypt foci were induced in Fischer’s rats using 1,2-dimethylhydrazine. This study revealed that black cumin oil potentially hindered post-initiation colon carcinogenesis [223].

Therefore, *N. sativa* is a promising natural medicinal candidate for the treatment of microbial infections, metabolic disorders, and other medical conditions. The pharmacological applications of this medicinal plant, with a focus on elucidating the mechanisms of action underlying its therapeutic effects, potential side effects, and existing clinical evidence supporting its use, are summarized in Table 5.

## 7. Future Perspectives

Expanding the study of bioactive substances found in *N. sativa* and their exceptional ability to inhibit microbial growth offers excellent prospects for advancing innovative agents with antimicrobial properties. Applying *N. sativa* plants as a cure for various microbial infections, metabolic disorders, and cancers provides a novel approach to effectively manage these medical conditions with the least possible side effects. Crucial progress toward potential pharmacological applications has been made by gaining insight into the antimicrobial effectiveness of *N. sativa* through in vivo experiments. In this context, a previous study explored the impact of *N. sativa* seeds on the colonization of intestinal *E. coli* and the morphology of the jejunal region in laying hens. One particular study focused on how *N. sativa* seeds influence the colonization of *E. coli* in the intestines and the overall structure of the jejunum in laying hens. The results showed that the application of 2% *N. sativa* led to optimal intestinal health by effectively reducing the presence of *E. coli* [255].

Moreover, research progress on the therapeutic role of *N. sativa* opens new prospects for scientists to discover more phytochemicals and naturally occurring compounds that may assist in treating disorders, subsequently offering better strategies to combat antimicrobial resistance, adverse effects of allopathic drugs, and other emerging challenges in disease management. A clinical study established that TQ effectively maintains the integrity of the intestinal barrier and hinders the translocation of bacteria. Administration of 10 mg of thymoquinone per kg body weight via the peritoneum in rats with intestinal obstruction, a condition associated with problems in movement and damage to the mucosal lining, significantly reduced oxidative stress and inflammatory cytokines. This protects the liver and intestines from inflammatory damage [256]. In another trial, combining 2 g of *N. sativa* with 40 mg of omeprazole proved highly effective in eradicating *Helicobacter pylori* within four weeks [257]. Recent nanotechnology advancements have enhanced the bioactivity of *N. sativa* compounds. Nanosuspensions improve solubility, bioavailability, and stability, leading to stronger antioxidant, antidiabetic, and antibacterial effects. These formulations offer a promising solution for targeted drug delivery and overcoming drug resistance [258].

Further research is needed to improve the efficacy of the use of plant extracts to combat infectious diseases and other health issues in terms of safety, long-term efficacy, and targeted therapy. If scientists overcome the shortcomings of these plant-based therapeutic interventions, the field of medicine will revolutionize and solve many of the major clinical problems currently faced by humanity around the world.

## 8. Conclusions

Our review clearly showed that *N. sativa* is a remarkable herb with great healing power owing to its unique chemical composition. This outstanding medicinal plant plays a vital role in the well-being of the human body by contributing to metabolic regulation, modulation of the immune function, and management of diverse infectious diseases. The use of black cumin seeds in routine food helps maintain homeostatic conditions in the body. Much research has been conducted on the clinical applications of *N. sativa* that has verified its great antimicrobial and therapeutic potential. This review highlighted promising avenues for harnessing the bioactivities of phytochemicals to bring novelty and improvement to the therapeutic management of medical conditions. Researchers and healthcare professionals continue to explore and harness the therapeutic potential of these compounds for a wide array of applications, highlighting the significance of *N. sativa* in the field of natural medicine. Further research and clinical studies are needed to fully realize the therapeutic benefits of this species and pave the way for the development of innovative and effective antimicrobial interventions.

## Figures and Tables

**Table 1:** Percentage yield of some solvent-extracted oils from *N. sativa* seeds of different countries [14].

Country	Yield %	Reference	Country	Yield %	Reference
Morocco	37	[15]	Egypt	34.8	[16]
Italy	13–23	[17]	Iran	40	[18]
Tunisia	28 31.7	[19]	Turkey	32 30–36	[20]
Pakistan	31.2	[21]	Bangladesh	32	[22]
Yemen	36.8–38.4	[23]	Saudi Arabia	38.2	[24]

**Table 2:** Key bioactive substances in *Nigella Sativa* and their importance: a comprehensive summary.

Compound Type	Bioactive Compounds	Activity Against Microorganism	Mode of Action	Reference
Quinones				
	Thymoquinone	*Staphylococcus aureus* (ATCC 9144)	Inhibition of bacterial cell wall synthesis	[122]
	Dithymoquinone	SARS-CoV-19	Inhibition of viral replication	[123]
Terpenes and Terpenoids				
	Alpha-hederin	EV71 subgenotypes C3 and C4a	Inhibition of viral replication	[124]
	Carvacrol	*Candida albicans*	Disruption of fungal cell membranes	[125]
	Thymol	Molds	Disruption of fungal cell membranes	[126]
	P-cymene	*Salmonella typhi* (ATCC 14028)	Inhibition of bacterial growth	[127]
	Camphene	*Candida albicans*	Disruption of fungal cell membranes	[128]
Alkaloids				
	Nigellidine	*Escherichia coli* (ATCC 25922)	Inhibition of bacterial enzymes	[129]
	Nigellimine	COVID-19	Inhibition of viral entry	[80]
	Nigellimine-N-oxide	NA	NA	NA
	Melanthin	Toxic	NA	NA
	Nigellidine	COVID-19	Inhibition of viral replication	[82]
	Nigellucin	NA	NA	NA
Fatty Acids and Sterols				
	Linoleic Acid	*Alternaria solani*, *Candida albicans*, *Crinipellis pernicosa*, *Fusarium oxysporum*, *Pyrenophora avanae*, *Pythium ultimum*, and *Rhizoctonia solani*	Disruption of fungal cell membranes	[130]
	Palmitic Acid	*Aspergillus flavus*	Disruption of fungal cell membranes	[131]
	Beta-sitosterol	Human Immunodeficiency Virus	Inhibition of viral entry	[132]
	Myristic Acid	NA	NA	NA
	Arachidonic Acid	*Staphylococcus aureus*	Lipid Peroxidation	[133]
	Oleic Acid	*Candida glabrata*	Disruption of fungal cell membranes	[134]
	Gamma-linolenic Acid	HIV	Destroys HIV-infected cells	[135]
Phenolic Compound				
	Eugenol	*Trichophyton rubrum*	Disruption of fungal cell membranes	[136]
Tocols				
	Alpha-Tocopherol	-	Scavenging free radicals	[137]
	Gamma-Tocopherol	-	Inhibition of pro-inflammatory mediators	[138]

**Table 3:** Toxicity profiles of bioactive compounds in *Nigella sativa*.

Sr. No.	Compound Name	PubChem CID	MolWeight (g/mol)	Mutagenic	Tumorigenic	Irritant
1	Nigellidine	136828302	294.3	N ^1^	N	N
2	Nigellimine	20725	203.24	N	N	N
3	Pentyl hexadec-12-enoate	74340768	324.5	Y ^2^	N	Y
4	Pentyl (*Z*)-pentadec-11-enoate	171120962	310.5	Y	N	Y
5	Lauric Acid	3893	200.32	Y	Y	Y
6	Myristic Acid	11005	228.37	Y	N	Y
7	Palmitic Acid	985	256.42	N	Y	Y
8	Vanillic acid	8468	168.15	Y	N	N
9	Epicatechin	72276	290.27	N	N	N
10	Quercetin	5280343	302.23	Y	Y	N
11	Apigenin	5280443	270.24	Y	N	N
12	3-Hydroxybenzoic acid	7420	138.12	N	N	N
13	Flavone	10680	222.24	Y	N	N
14	Myricetin	5281672	318.23	Y	N	N
16	Naringenin	439246	272.25	Y	N	N
17	Kaempferol	5280863	286.24	Y	N	N
18	Chrysin	5281607	254.24	N	N	N
19	Pinocembrin	68071	256.25	N	N	N
20	Galangin	5281616	270.24	Y	N	N
21	Camphene	6616	136.23	Y	N	N
22	Linalool	6549	154.25	Y	N	Y
23	Camphor	2537	152.23	Y	Y	Y
24	Nerol	643820	154.25	N	N	N
25	Carvone	7439	150.22	Y	Y	Y
26	Thymoquinone	10281	164.2	Y	N	N
27	Umbellulone	442504	150.22	N	N	N
28	Carvacrol	10364	150.22	N	N	Y
29	Longifolene	289151	204.35	N	N	N
30	Cyclosativene	519960	204.35	N	N	N
31	Aromadendrene	91354	204.35	N	Y	Y
32	Myrcene	31253	136.23	N	Y	Y
33	p-CYMENE	7463	134.22	N	Y	Y
34	Limonene	22311	136.23	Y	Y	Y
35	Terpinolene	11463	136.23	N	N	N
36	Citronellyl acetate	9017	198.3	N	N	Y
37	Thymohydroquinone	95779	166.22	Y	Y	N
38	Tricyclene	79035	136.23	N	N	N
39	Borneol	1201518	154.25	Y	N	Y
40	Myrtenol	10582	152.23	N	N	Y
41	Cuminaldehyde	326	148.2	N	N	Y
42	Bornyl acetate	6448	196.29	N	N	Y
43	Thymol	6989	150.22	Y	N	N
44	Methyl geranate	5365910	182.26	Y	N	Y
45	Neryl acetate	1549025	196.29	Y	Y	Y
46	Sabinene	18818	136.23	N	N	N
47	Estragole	8815	148.2	Y	Y	Y
48	Myristicin	4276	192.21	N	N	Y
49	Apiole	10659	222.24	Y	N	N
50	Eugenol	3314	164.2	N	N	N
51	Dodecanal	8194	184.32	Y	N	Y
52	Benzaldehyde	240	106.12	Y	Y	Y
53	Coumarin	323	146.14	Y	Y	N
54	Tetradecanal	31291	212.37	Y	N	Y
55	Methyl linoleate	5284421	294.5	N	N	N

^1^ No, ^2^ Yes.

**Table 4:** Drug-likeness and pharmacokinetic properties of bioactive compounds in *Nigella sativa*.

Sr. No.	Compound Name	cLogP	Solubility	TPSA	Drug-Likeness	Drug Score
1	Nigellidine	1.54	−2.29	43.8	1.51	0.83
2	Nigellimine	2.11	−2.83	31.4	−0.42	0.64
3	Pentyl hexadec-12-enoate	8.01	−5.25	26.3	−27.08	0.09
4	Pentyl (*Z*)-pentadec-11-enoate	7.55	−4.98	26.3	−27.06	0.08
5	Lauric Acid	4.24	−3.16	37.3	−25.22	0.08
6	Myristic Acid	5.15	−3.7	37.3	−25.22	0.12
7	Palmitic Acid	6.06	−4.24	37.3	−25.01	0.09
8	Vanillic acid	0.73	−1.35	66.8	−1.31	0.35
9	Epicatechin	1.51	−1.76	110	1.92	0.87
10	Quercetin	1.49	−2.49	127	1.6	0.3
11	Apigenin	2.34	−2.86	87	1.21	0.47
12	3-Hydroxybenzoic acid	0.8	−1.33	57.5	−4.27	0.3
13	Flavone	3.37	−3.74	26.3	1.85	0.45
14	Myricetin	1.14	−2.2	147	0.75	0.46
16	Naringenin	2.16	−2.64	87	1.9	0.51
17	Kaempferol	1.84	−2.79	107	0.9	0.46
18	Chrysin	2.68	−3.15	66.8	0.97	0.75
19	Pinocembrin	2.5	−2.94	66.8	1.95	0.83
20	Galangin	2.18	−3.08	87	0.66	0.44
21	Camphene	2.8	−2.69	0	−5.86	0.27
22	Linalool	3.23	−2.15	20.2	−6.68	0.16
23	Camphor	2.18	−2.45	17.1	−3.71	0.06
24	Nerol	3.49	−1.89	20.2	−3.57	0.45
25	Carvone	2.65	−2.19	17.1	−18.99	0.1
26	Thymoquinone	1.64	−1.68	34.1	−1.2	0.35
27	Umbellulone	2.07	−2.17	17.1	−2.81	0.5
28	Carvacrol	2.84	−2.53	20.2	−2.59	0.29
29	Longifolene	4.06	−3.81	0	−7.76	0.37
30	Cyclosativene	3.71	−3.77	0	−6.86	0.39
31	Aromadendrene	4	−3.79	0	−7.14	0.14
32	Myrcene	4.29	−2.5	0	−7.82	0.09
33	p-CYMENE	3.19	−2.83	0	−5.63	0.21
34	Limonene	3.36	−2.54	1.7	−21.85	0.06
35	Terpinolene	3.45	−2.34	1.9	−3.02	0.46
36	Citronellyl acetate	3.83	−2.56	26.3	−4.29	0.25
37	Thymohydroquinone	2.5	−2.24	40.5	−6.33	0.22
38	Tricyclene	2.45	−2.67	0	−2.38	0.5
39	Borneol	2.04	−2.4	20.2	−3.53	0.17
40	Myrtenol	1.79	−2.01	20.2	−1.56	0.33
41	Cuminaldehyde	2.78	−2.81	17.1	−11.1	0.27
42	Bornyl acetate	2.52	−2.81	26.3	−3.69	0.28
43	Thymol	2.84	−2.53	20.2	−3.02	0.17
44	Methyl geranate	3.56	−2	26.3	−10.38	0.21
45	Neryl acetate	3.97	−2.3	26.3	−2.88	0.1
46	Sabinene	2.86	−2.69	0	−6.78	0.45
47	Estragole	2.62	−2.35	9.23	−3.75	0.1
48	Myristicin	2.73	−3.06	27.7	−2.29	0.17
49	Apiole	2.66	−3.08	36.9	−4.67	0.27
50	Eugenol	2.27	−2.05	29.5	−2.78	0.11
51	Dodecanal	4.38	−3.39	17.1	−22.31	0.08
52	Benzaldehyde	1.59	−1.94	17.1	−4.05	0.11
53	Coumarin	1.5	−2.37	26.3	−1.83	0.12
54	Tetradecanal	5.29	−3.93	17.1	−22.3	0.07
55	Methyl linoleate	6.89	−4.45	26.3	−35.73	0.22

**Table 5:** An overview of the pharmacological applications of *Nigella sativa*.

Pharmacological Application	Formulation	Mechanism of Action	Potential Side Effects	Clinical Evidence	References
Anti-inflammatory Effects	*Nigella sativa* oil or extract	Inhibition of inflammatory pathways, such as NF-κB, by active component thymoquinone.	Limited reports of gastrointestinal discomfort.	Some studies support anti-inflammatory effects in various conditions.	[141,143,198,224]
Antioxidant Properties	*Nigella sativa* seed extract or oil	Presence of antioxidants (e.g., thymoquinone) to neutralize free radicals and reduce oxidative stress.	Generally well tolerated; rare reports of allergic reactions.	Evidence supports antioxidant properties in vitro and in animal studies.	[79,141,143,225]
Antimicrobial Activity	*Nigella sativa* seed oil	Thymoquinone exhibits antimicrobial properties, disrupting cell membranes and inhibiting protein synthesis.	Limited reports of skin irritation; caution in pregnant women.	Limited clinical evidence; more research is needed for specific infections.	[147,226,227,228]
Anticancer Potential	*Nigella sativa* oil or thymoquinone	Induction of apoptosis, inhibition of cell proliferation, and anti-inflammatory effects.	Limited studies; potential interactions with cancer treatments.	Some promising preclinical evidence; more research is needed in humans.	[75,229]
Immunomodulatory Effects	*Nigella sativa* extract	Modulation of the immune system, enhancing activity of immune cells (e.g., T cells and natural killer cells), and regulating cytokine production.	Rare reports of allergic reactions; caution in autoimmune diseases.	Limited clinical evidence; potential benefits observed in some studies.	[175,230,231,232,233]
Cardioprotective Effects	*Nigella sativa* oil or extract	Reduction of oxidative stress, inflammation, and improvement of lipid profiles.	Generally well tolerated; caution in individuals with bleeding disorders.	Positive effects on cardiovascular risk factors were observed in some studies.	[44,234,235]
Antidiabetic Effects	*Nigella sativa* seed powder or oil	Potential hypoglycemic effects, improving insulin sensitivity and reducing complications associated with diabetes.	Caution in hypoglycemic individuals: monitor blood sugar levels.	Some studies suggest improved glycemic control; more research is needed.	[76,231,236,237,238]
Neuroprotective Properties	*Nigella sativa* oil or extract	Neuroprotective effects by reducing oxidative stress, inflammation, and apoptosis in certain neurological conditions.	Generally well tolerated; caution in individuals with neurological disorders.	Positive effects observed in preclinical models; limited human studies.	[79,212,232,239,240]
Respiratory Health	*Nigella sativa* essential oil	Potential bronchodilator effects, easing symptoms of asthma and improving respiratory function.	Limited reports of throat irritation; avoid in excessive amounts.	Limited clinical evidence; more research is needed for respiratory conditions.	[232,241,242]
Gastrointestinal Health	*Nigella sativa* seed powder	Gastroprotective effects promote the healing of gastric ulcers and reduce gastrointestinal disorder symptoms.	Rare reports of nausea and bloating; consult with a healthcare professional.	Some evidence for gastroprotective effects in animal studies.	[238,243,244]
Skin Health	*Nigella sativa* oil or cream	Anti-inflammatory and antioxidant properties may benefit skin conditions, such as eczema and psoriasis.	Patch test recommended; rare reports of skin irritation.	There is limited clinical evidence; more research is needed for specific skin conditions.	[245,246,247,248,249,250]
Reproductive Health	*Nigella sativa* supplementation	Potential fertility benefits in both men and women, may regulate menstrual cycles and improve sperm parameters.	Consultation with a healthcare provider is advised, especially during pregnancy.	Limited clinical evidence; more research is needed for fertility outcomes.	[251,252,253,254]

## Data Availability

Data is contained within the article.
